# Between a rock and a hard place: anticoagulation management for ECMO

**DOI:** 10.1007/s00063-024-01116-0

**Published:** 2024-03-08

**Authors:** Nina Buchtele, Jerrold H Levy

**Affiliations:** 1 Intensive Care Unit 13i2, Department of Medicine I, Medical University of Vienna, Währinger Gürtel 18–20, 1090 Vienna, Austria; 2 Department of Anesthesiology, Critical Care, and Surgery (Cardiothoracic), Duke University School of Medicine, Durham, NC USA

**Keywords:** Anticoagulants, Extracorporeal membrane oxygenation, Thrombosis, Thromboembolism, Hemorrhage, Antikoagulation, Extrakorporale Membranoxygenierung, Gerinnung, Thrombose, Blutung

## Abstract

Anticoagulation is an essential component of optimal extracorporeal membrane oxygenation (ECMO) management. Unfractionated heparin is still the anticoagulant of choice in most centers due to longstanding familiarity with the agent. Disadvantages include alterations in drug responses due to its capability to bind multiple heparin-binding proteins that compete with antithrombin and the potential for heparin-induced thrombocytopenia. In such cases, direct thrombin inhibitors are the treatment of choice but pose difficulties in monitoring due to the limited experience and target ranges for non-aPTT-guided management (aPTT: activated partial thromboplastin time). The current trend toward low-dose anticoagulation, especially for venovenous ECMO, is supported by data associating bleeding complications with mortality but not thromboembolic events, which include circuit thrombosis. However, only prospective data will provide appropriate answers to how to individualize anticoagulation, transfusions, and bleeding management which is currently only supported by expert opinion. Empiric therapy for ECMO patients based on laboratory coagulation alone should always be critically questioned. In summary, only collaboration and future studies of coagulation management during ECMO will help us to make this life-saving therapy that has become part of daily life of the intensivist even safer and more effective. Until then, a fundamental understanding of coagulation and bleeding management, as well as pearls and pitfalls of monitoring, is essential to optimize anticoagulation during ECMO. This article is freely available.

## Abstract (de)

Die Antikoagulation ist ein wesentlicher Bestandteil eines optimalen Managements der extrakorporalen Membranoxygenierung (ECMO). Unfraktioniertes Heparin ist in den meisten Zentren immer noch das Antikoagulans der Wahl, zum Teil auch aufgrund seiner langjährigen Verwendung und Vertrautheit. Zu den Nachteilen gehören Veränderungen im Ansprechen auf das Medikament aufgrund seiner Fähigkeit, mehrere heparinbindende Proteine zu binden, die mit Antithrombin um dessen Bindung konkurrieren. Außerdem kann Heparin eine Heparin-induzierte Thrombozytopenie verursachen. In solchen Fällen sind direkte Thrombininhibitoren die Antikoagulanzien der Wahl, die jedoch bei einem nicht durch die aktivierte partielle Thromboplastinzeit (aPTT) gesteuerten Management aufgrund der begrenzten Erfahrung und fehlender Zielbereiche schwierig zu überwachen sind. Der derzeitige Trend zu einer niedrigdosierten Antikoagulation, insbesondere bei Patient:innen mit venovenöser ECMO, wird durch Daten gestützt, die einen Zusammenhang zwischen Blutungskomplikationen und Sterblichkeit zeigen, nicht aber bei thromboembolischen Ereignissen, die meist als Thrombosen des extrakorporalen Kreislaufs evident werden. Dennoch können nur prospektive Daten angemessene Antworten darauf geben, wie die Antikoagulation tatsächlich individualisiert werden könnte. Das Gleiche gilt für Transfusionen und die Verabreichung von gerinnungsaktiven Substanzen, die derzeit nur durch Expert:innenmeinungen gestützt wird. Eine empirische Therapie für ECMO-Patient:innen, die sich allein auf Labortests stützt, sollte daher immer kritisch hinterfragt werden. Zusammenfassend lässt sich sagen, dass nur eine Zusammenarbeit und zukünftige Studien zum Gerinnungsmanagement während der ECMO dazu beitragen können, diese lebensrettende Therapie, die heute zur täglichen Routine der Intensivmediziner:innen gehört, noch sicherer und effektiver zu gestalten. Bis dahin ist ein grundlegendes Verständnis der Gerinnung und gerinnungsaktiven Substanzen sowie der Schwierigkeiten im Monitoring unerlässlich, um die Antikoagulation während der ECMO zu optimieren. Dieser Artikel ist frei verfügbar.

## Introduction

Extracorporeal membrane oxygenation (ECMO) provides cardiopulmonary support using venoarterial (VA) ECMO or lung support with venovenous (VV) ECMO. During ECMO, blood interfaces with the nonendothelialized tubing and membrane oxygenation, activating coagulation. As a result, ECMO requires anticoagulation [1] to prevent thrombosis and thromboembolism in intensive care unit (ICU) patients. The coagulopathy in ECMO is complex due to high shear forces, hemostatic activation, clotting factor consumption, and platelet activation [2]. The challenge for ICU physicians is to balance the dysregulated coagulation system of thromboembolism and bleeding with appropriate anticoagulation and other potential therapies.

## Which anticoagulant is optimal?

Currently, there is no evidence-based answer for optimal anticoagulation, including center-specific experience and anticoagulant availability. Current agents include unfractionated heparin (UFH) and direct thrombin inhibitors (DTIs) [1], although adjunct platelet-modifying agents are also reported [3, 4]. Use of factor XI (FXI)- and factor XII (FXII)-targeting agents are still under investigation.

### Heparins—old but gold?

Unfractionated heparin binds to antithrombin (AT), including both factor Xa (FXa) and factor IIa (FIIa, thrombin), to provide its anticoagulant effect [5]. Unfractionated heparin inhibits in a 1:1 ratio, as smaller low molecular weight heparin (LMWH) molecules inhibit FXa.

According to a recent survey, UFH is the anticoagulant in 96% of ECMO centers [6]. The dose-response to UFH varies, requiring more frequent coagulation monitoring [7]. Unfractionated heparin binds to multiple plasma proteins beyond AT, including acute-phase-reactant proteins (e.g., heparin cofactor II), that also contribute to antiinflammatory effects [8]. However, multiple heparin-binding proteins compete with AT for binding, including vitronectin, which contributes ~50% of UFH binding in sepsis, and heparin resistance [8]. Despite initial older reports arbitrarily suggesting > 35,000 IU/day UFH for heparin resistance, a recent literature review and survey including coronavirus disease 2019 (COVID 19) patient data reported current definitions include weight-adapted dosing and suggested this is not resistance or failure to anticoagulate but rather alterations in drug responses that depend on testing used [9].

Heparin is also the cause of heparin-induced thrombocytopenia (HIT; type I and II). Heparin-induced thrombocytopenia I defines nonimmune effects that decrease platelet to ~100 G/L, commonly after ECMO initiation [10], and without thrombosis. Heparin-induced thrombocytopenia II is an immune response from IgG antibodies to platelet factor 4 (PF4)/heparin complexes and exposure to heparin. The IgGs to PF4 on platelets’ surfaces activate and aggregate platelets, causing thrombosis [11]. Heparin-induced thrombocytopenia II occurs in < 5% of ECMO patients anticoagulated with UFH [12].

### Direct thrombin inhibitors—a better choice?

Bivalirudin and argatroban are parenteral direct thrombin inhibitors (DTIs) inhibiting circulating and bound thrombin [13]. A recent meta-analysis comparing UFH with DTIs regarding thromboembolic and bleeding complications reported DTI-treated patients had fewer major bleeding events and pump-related thrombosis. However, this result is from retrospective cohort studies where UFH was switched to a DTI, which limit interpretation.

Whether DTIs for ECMO improve outcomes remain to be determined, and are exceedingly difficult to evaluate as the underlying disease process requiring ECMO primarily determines outcomes [14], but are the mainstay therapy for HIT [11], due to the lack of DTI cross-reactivity with PF4 antibodies that induce HIT II [14]. There is no antidote available; however, prothrombin complex concentrate (PCC), recombinant factor VIIa, and factor VIII have been evaluated in vitro to restore coagulation [13, 15]. Since DTIs have a short half-life, whether immediate reversal is needed during ECMO with the further inherent risk of thrombosis remains to be determined.

### Broadening the spectrum—platelet-targeting agents

Although platelets are an important component for hemostasis, platelet inhibitors have been studied as adjuncts for anticoagulation alone. A VV ECMO trial reported that prostaglandin E1 at 5 ng/kg/min in addition to UFH reduced complications [4]. In pumpless arteriovenous interventional lung assist, additional low-dose aspirin (1.5 mg/kg/day) preserved oxygenator performance without increasing bleeding complications compared to UFH alone [3].

Patients requiring (VA) ECMO because of acute MI triggered cardiogenic shock after PCI and stent implantation require dual antiplatelet therapy and anticoagulation. Optimal management of these patients for safety or efficacy for a specific P2Y12 inhibitor (prasugrel, ticagrelor, or cangrelor) is currently unknown [16]. These patients are at increased risk of bleeding (~75%) and stroke and should be a specific subgroup for future trials.

## Keeping track—pearls and pitfalls in anticoagulation monitoring

### Basics of coagulation tests

The principles of the available coagulation tests are important to understand for anticoagulation monitoring. Coagulation tests differ by (I) the material tested, (II) the activator of coagulation and the final product measured, as well as (III) the measurement method used. Depending on these factors, the various coagulation tests reflect different aspects of coagulation that can be influenced by multiple confounders (Table 1).

I) Coagulation parameters can be measured in (citrated) whole blood or plasma. To perform plasma-based tests, centrifugation is required to separate the cellular components of the blood. Consequently, unlike whole blood-based tests, plasma-based tests do not reflect the (I) cellular components of the coagulation cascade and (II) are not available as bedside methods.
II) The measured assay endpoint and the activator used determines the value based on seconds for a clot-based coagulation test that use fibrin formation. In this context, the assessment is influenced by factors beyond anticoagulation that affect clot formation, including hyperfibrinolysis [17], or factors affecting fibrin polymerization, such as paraproteinemia [18].
III) Clinical measurement methods are used to determine the corresponding end product: coagulometric detection (’clot-based’), chromogenic, and oscillometric measurements. The first two are photo-optical methods, i.e., measurements of color changes. On the other hand, oscillometry is a mechanical measurement method used in viscoelastic testing, where an oscillating pin or cup is restricted in its mobility by an increase in clot strength [19]. Methods based on color detection, especially chromogenic measurements, are affected by conditions that alter the native color of plasma, such as hyperbilirubinemia or hemolysis [20].

**Table 1:** Overview of coagulation tests

		Test	Activator	End point	Measurement method	Measured section	Specific confounders
Plasma-based tests		aPTT	Ellagic acid, kaolin, silica, celite	Clot formation	Clot-based (coagulometric)	Contact activation (intrinsic) pathway	Lupus anticoagulant, decreased FXII activity, high FVIII activity, hyperfibrinolysis
		AntiXa	Factor Xa	Color change (Residual Factor Xa)	Chromogenic	Factor Xa activity	Hyperbilirubinemia, hemolysis
			Factor Xa	Clot formation	Clot-based (coagulometric)	Common pathway	Hyperfibrinolysis
		Diluted thrombin time (Hemoclot assay)	Thrombin (Factor IIa)	Clot formation	Clot-based (coagulometric)	Common pathway	Hypoalbuminemia
		Ecarin chromogenic assay	Ecarin	Color Change (Residual factor IIa)	Chromogenic	FIIa activity	Hyperbilirubinemia, hemolysis
Whole-blood-based tests	–	Activated clotting time	Kaolin, celite	Clot formation	Clot based (coagulometric)	Contact activation (intrinsic) pathway	Hypothermia, hypovolemia, hyperfibrinolysis, thrombocytopenia, factor deficiency
	Viscoelastic tests	Intem	Ellagic Acid	Clot formation	Change in oscillation	Contact activation (intrinsic) pathway	Interuser variability
		CK	Kaolin				
		Heptem	Ellagic acid + heparinase	Clot formation (neutralizing heparin effect)	Change in oscillation	Contact activation (intrinsic) pathway	
		CKH	Kaolin + heparinase				
		Fibtem	Tissue factor + heparinase + cytochalasin	Clot formation (neutralizing platelets)	Change in oscillation	Tissue-factor activated (extrinsic) pathway	
		Functional fibrinogen	Tissue factor + abciximab				

*aPTT* activated partial thromboplastin time, *CK* citrated kaolin, *CKH* citrated kaolin with heparinase

The optimal single monitoring test has not yet been determined. Present data suggest that anti-Xa assays correlate best with UFH dosing [21]. Titration according to antiXa levels is therefore recommended by the current International Society on Thrombosis and Haemostasis (ISTH) guidelines [1]. AntiXa assays have test-specific differences with respect to the measurement method (coagulometric or chromogenic; relevant for interfering factors) and possible in vitro AT compensation. Therefore, consultation with the local laboratory physician is essential for the correct interpretation of the test results.

For DTIs, there is currently insufficient experience with monitoring methods that more specifically map the thrombin inhibitory effect (Hemoclot assay and ecarin clotting time), analogous to antiXa for UFH [22]. For lack of appropriate reference ranges for anticoagulation on ECMO, aPTT-guiding is the currently recommended assay [1].

However, the coagulation system complexity must be considered, as global plasma-based coagulation assays and viscoelastic tests may better characterize coagulopathies and bleeding management.

## A balancing act—intensity of anticoagulation

Patients differ in many domains that influence coagulation. With the increasing ECMO use clinically, a one-size-fits-all approach to anticoagulation strategies for these patients may not be appropriate. However, the fundamental goal of anticoagulation that intensivists should consider is safe and effective therapy to prevent thrombotic complications, maintain ECMO circuit patency, and minimize bleeding complications.

Ongoing technological improvements in the hemocompatibility of the ECMO circuit, including pump drive optimization (centrifugal pumps instead of roller pumps) and coating improvement for blood surface interfaces have facilitated lower-dose anticoagulation to maintain circuit patency [23]. This is important for VV ECMO patients requiring respiratory support as thrombotic complications can initially manifest as ECMO oxygenator membrane clotting impairing extracorporeal gas exchange. Blood gas analyses at sampling points after the oxygenator reveal deteriorating efficacy at low thresholds before any detectable worsening of the patient’s gas exchange. Consequently, if necessary, the circuit can be changed (semi-)electively—in < 15 s with optimal preparation. Emboli from the membrane thrombotic source deposit in the lung with VV cannulation, often with minimal consequences. A low-dose anticoagulation approach is supported by a recent nationwide French COVID-19 registry study of mostly VV ECMO patients reporting bleeding complications were associated with increased mortality but not thrombotic events [24].

In VA ECMO patients, small thromboemboli can have disastrous consequences, including disabling strokes and limb ischemia, as the emboli distribute throughout the arterial system. Whether prevention of these complications is best accomplished by intensifying anticoagulation, adding platelet aggregation inhibitors, or both is unknown, but adding additional platelet inhibition presents with increased bleeding risk. However, numerous data report that the risk for complications increases with longer ECMO duration, especially in VA ECMO patients [25, 26].

Further, scientific support for an optimal individualized anticoagulation strategy to better assess individual thrombosis and bleeding risk is unknown. While large registry studies have identified low pH, ventilation days, and acute kidney injury at cannulation as risk factors for complications [24–26], a closer look at coagulation parameters, especially during ECMO, may further help risk stratification [17].

## Liberal or restrictive—transfusions and antithrombin

In addition to anticoagulation, other options are available to the intensivist to optimize coagulation. However, any therapeutic decision should include careful benefit/risk assessment.

### Platelets

While it is recommended to monitor platelet counts during ECMO, no studies are available to guide platelet transfusions from an evidence-based perspective [1]. Most centers use a threshold of 50 G/L for platelet administration in nonbleeding patients [18] and are in agreement with Extracorporeal Life Support Organization (ELSO) guidelines [7]. Although thrombocytopenia contributes to bleeding during ECMO, only the absolute platelet count has been considered without platelet function monitoring (PFM) or considerations of changes over time [27]. Ongoing evaluation of PFM is needed to be applied in clinical management.

### Antithrombin

One of the very few prospective, controlled trials of ECMO randomized 48 patients to receive routine AT supplementation to achieve > 80% AT III activity or placebo [28]. This well-performed study suggests that AT supplementation does not decrease UFH requirement or thrombotic or bleeding complications. However, mild AT deficiency is reported to increase thromboembolic risk [29]. Whether routine AT supplementation should be performed at lower thresholds (< 50%) or whether these patients would benefit from AT-independent anticoagulation with DTIs remains to be determined.

### Fresh frozen plasma and prothrombin complex concentrate

The ELSO guidelines on anticoagulation suggest fresh frozen plasma (FFP) administration if the international normalized ratio (INR) > 1.5 with bleeding and routine supplementation if INR > 3 as an expert opinion [7]. However, a recent study reported FFPs are administered to ~60% of VA ECMO and ~40% of VV ECMO patients, of which only 40% met ELSO-suggested thresholds [30]. Although less frequent, some centers use prothrombin complex concentrate (PCC) for bleeding management [30], which is not mentioned in current guidelines. Given the lack of prospective studies, algorithms used in postoperative and critically ill patients without ECMO are currently used.

### Fibrinogen and antifibrinolytics

Guidelines suggest maintaining fibrinogen > 100 mg/dl in the nonbleeding, and > 150 mg/dl in the bleeding ECMO patient [7]. However, most current recommendations in bleeding patients suggest targeting higher fibrinogen levels of at least 200 mg/dL (2 g/L). For further evaluation, examination of the ECMO oxygenator facilitates potential detection of membrane clotting, viscoelastic tests to detect hyperfibrinolysis, disseminated intravascular coagulation screening with lab tests, and analysis of liver function tests should be considered in the coagulopathic patient. While a circuit exchange can resolve localized membrane clotting, patients with generalized hyperfibrinolysis may benefit from antifibrinolytic agents (tranexamic acid) [31]. Lower thresholds for fibrinogen repletion may be considered in nonbleeding patients with liver dysfunction.

In summary, a more comprehensive (laboratory) diagnostic workup is needed in ECMO patients with active bleeding to optimize therapeutic interventions. Routine transfusions and administration of procoagulant products to manage bleeding complications must be balanced against the potential prothrombotic effects on the circuit, and may depend on the underlying medical condition that require the use of ECMO.

## The journey continues from eminence- to evidence-based strategies of anticoagulation

Although anticoagulation is essential to ECMO management, and publications on this topic are growing steadily, many fundamental questions discussed in this review cannot be answered from an evidence-based perspective.

As a reminder, increasing literature reports of anticoagulation in ECMO does not necessarily mean increasing quality. Despite the increasing use of ECMO, it is surprising how few high-quality randomized controlled trials have been published or are currently being conducted (Table 2). The few published studies include only small numbers of cases and do not reflect the increasing ECMO use. International collaborations to conduct prospective studies often fail because of center-specific differences in anticoagulation regimens and standard operating procedures (SOPs) that limit comparability.

**Table 2:** Overview of ongoing interventional trials assessing a) different anticoagulants and b) different intensity of anticoagulation. c) Other interventional trials regarding anticoagulation

Clinicaltrials.gov identifier	Intervention	Study design	Participants	ECMO type	Outcome	Trial site	Study start	Study completion
*a) Interventional trials assessing different agents for anticoagulation in adults*								
NCT04496362	LMWH vs. UFH	Randomized, open-label	100	VV	Bleeding, thrombosis	Baylor Scott & White Health research institute (Dallas, TX USA)	10/2018	12/2023
NCT04536272	UFH high dose (aPTT 60–75 s) vs. UFH low dose (aPTT 45–60 s) vs. LMWH (weight and renal function adjusted)	Randomized, open-label	330	VA & VV	Bleeding, thrombosis, mortality	Multicenter, Responsible Party: University Medical Center Groningen (Groningen, Netherlands)	10/2020	03/2024
NCT05959252	Bivalirudin vs. UFH	Randomized, open-label	80	VV & VA	Feasibility (time in therapeutic range)	Royal Prince Alfred Hospital (Sydney, Australia)	09/2023	05/2026
NCT03965208	Bivalirudin vs. UFH	Randomized, open-label	34	VA & VV	Feasibility (anticoagulation targets met)	Legacy Emanuel Medical Center (Portland, OR USA)	05/2019	Unknown
NCT04925167	Argatroban vs. UFH	Randomized, open-label	174	VV	Thrombosis	Beijing Chao Yang Hospital (Beijing, China)	07/2021	06/2024
NCT05226442	Argatroban vs. UFH	Randomized, open-label	40	VA & VV	Bleeding, thrombosis	Medical University of Vienna (Vienna, Austria)	12/2021	06/2024
NCT05555641	Nafamostat Mesylate vs. UFH	Randomized, open-label	40	VA & VV	Bleeding	Wuhan Union Hospital (Hubei, China)	12/2022	10/2024
*b) Interventional trials assessing different intensity of anticoagulation in adults*								
NCT04997265	Prophylactic vs. moderate intensity UFH (AntiXa 0.2–0.3 IU/mL)	Randomized, open-label	15	VV	Feasibility (recruitment)	Vanderbilt University Medical Center (Nashville, TN USA)	05/2022	05/2024
NCT04273607	Prophylactic vs. standard of care (UFH)	Randomized, open-label	40	VV	ECMO-associated thrombosis	Toronto General Hospital (Toronto, ON Canada)	09/2022	06/2024
NCT05697692	No anticoagulation vs. UFH during Lung transplantation	Randomized, double-blind, placebo controlled	80	Central VA	Thrombosis	Medical University of Vienna (Vienna, Austria)	12/22	06/24
*c) Other interventional trials in adults*								
NCT05239637	UFH Stop 1 h before removal vs. within 24 h after	Randomized, blinded outcome assessment	40	VA & VV	Bleeding, thrombosis	Zhengzhou University (Henan, China)	02/2022	10/2023
NCT05148026	Regional citrate anticoagulation for CRRT	Randomized, cross-over, open-label	20	VV	CRRT circuit clotting	University of Milano Bicocca (Monza, Italy)	11/2021	12/2023
NCT03261284	D‑dimer/AntiXa guided anticoagulation vs. AntiXa/aPTT guided	Randomized, open-label	300	VA & VV	Bleeding, thrombosis	Wuhan Asia Heart Hospital, Wuhan, China	03/2019	12/2023
NCT03613584	Von Willebrand factor	Randomized, placebo-controlled, double-blind	68	VA & VV	PRBC transfusion requirement	Medical University of Innsbruck (Innsbruck, Austria)	04/2018	Unknown

*aPTT* activated partial thromboplastin time; *CRRT* continuous renal replacement therapy; *ECMO* extracorporeal membrane oxygenation; *LMWH* low-molecular weight heparin; *UFH* unfractionated heparin; *PRBC* packed red blood cell; *VA* venoarterial; *VV* venovenous

Another challenge is the definition of clinically relevant endpoints. Whether hemorrhage affects only the cannula puncture site or whether a hematothorax requiring surgery has occurred requires some differentiation in reporting. Therefore, comparing outcomes without a uniform classification of bleeding complications under ECMO therapy is difficult. In this context, grading of the severity of hemorrhage is essential. To optimize classification, we could learn from interventional, cardiovascular trials: to harmonize bleeding classifications used in these clinical trials, the Bleeding Academic Research Consortium proposed a standardized bleeding definition for reporting adverse events [32].

Similarly, thrombotic complications should also be reported in a nuanced manner. For example, an elective, uncomplicated circuit change for thrombosis should be considered differently from a major stroke with hemiparesis. In this context, the global ELSO registry provides robust mortality data of large numbers of cases, but lacks differences in collecting clinically relevant endpoints. More detailed data entries, in turn, take more time, resulting in a financial issue.

Although the number of ongoing studies in the context of anticoagulation during ECMO is limited, they will hopefully provide additional insight into clinical management. Further, the challenge of doing these clinical studies is the underlying disease process that necessitated ECMO use is a major factor in determining important outcomes including mortality. The way forward, however, as the global COVID-19 pandemic has already taught us, will inevitably require closer international collaboration to achieve appropriate sample sizes to answer important questions about coagulation management.

## Practical conclusion

Anticoagulation is an essential component of optimal extracorporeal membrane oxygenation (ECMO) management. While unfractionated heparin (UFH) is currently the anticoagulant of choice, prospective studies will hopefully evaluate objective data for direct thrombin inhibitors (DTIs) or additional studies for antithrombin (AT) supplementation. A basic understanding of coagulation and available tests to monitor anticoagulation is essential to decide which test is useful to titrate the anticoagulant and which tests can help manage bleeding and prevent thromboembolic events. Also, only prospective studies will demonstrate whether the trend toward low-dose anticoagulation is safe and effective. Empiric therapy for ECMO patients based on laboratory coagulation alone by transfusions and coagulation-active substances should always be critically questioned while no high-quality studies are available and supported currently by expert opinion. In summary, only collaboration and cooperation in the coagulation management during ECMO will help us to make this life-saving therapy, which has now become part of daily life of the intensivist, even safer and more effective.

## Declarations

For this article no studies with human participants or animals were performed by any of the authors. All studies mentioned were in accordance with the ethical standards indicated in each case.

The supplement containing this article is not sponsored by industry.
